# A Novel Polyvinylidene Fluoride Tree-Like Nanofiber Membrane for Microfiltration

**DOI:** 10.3390/nano6080152

**Published:** 2016-08-19

**Authors:** Zongjie Li, Weimin Kang, Huihui Zhao, Min Hu, Na Wei, Jiuan Qiu, Bowen Cheng

**Affiliations:** 1 College of Textile, Tianjin Polytechnic University, Tianjin 300387, China; lpllzj@126.com (Z.L.); zhaohuihuitjpu@126.com (H.Z.); humin.1990@163.com (M.H.); 2 College of Packaging and Printing Engineering, Tianjin Vocational Institute, Tianjin 300387, China; weina209@163.com (N.W.); qiujiuan@126.com (J.Q.); 3 State Key Laboratory of Separation Membranes and Membrane Processes, Tianjin Polytechnic University, Tianjin 300387, China

**Keywords:** electrospinning, polyvinylidene fluoride, tree-like, nanofibers, microfiltration

## Abstract

A novel polyvinylidene fluoride (PVDF) tree-like nanofiber membrane (PVDF-TLNM) was fabricated by adding tetrabutylammonium chloride (TBAC) into a PVDF spinning solution via one-step electrospinning. The structure of the prepared membranes was characterized by field emission scanning electron microscopy (FE-SEM), Fourier transform infrared spectroscopy (FT-IR) and pore size analysis, and the hydrophilic property and microfiltration performance were also evaluated. The results showed that the tree-like nanofiber was composed of trunk fibers and branch fibers with diameters of 100–500 nm and 5–100 nm, respectively. The pore size of PVDF-TLNM (0.36 μm) was smaller than that of a common nanofiber membrane (3.52 μm), and the hydrophilic properties of the membranes were improved significantly. The PVDF-TLNM with a thickness of 30 ± 2 μm showed a satisfactory retention ratio of 99.9% against 0.3 μm polystyrene (PS) particles and a high pure water flux of 2.88 × 10^4^ L·m^−2^·h^−1^ under the pressure of 25 psi. This study highlights the potential benefits of this novel PVDF tree-like nanofiber membrane in the membrane field, which can achieve high flux rates at low pressure.

## 1. Introduction

Recently, with the rapid development of the economy, the worldwide problems associated with the shortage of clean, fresh water have attracted more and more attention [1,2,3]. Over one billion people do not have access to clean water and over two billion people live in water-stressed areas [4]. Membrane technology is one of the most important and versatile technologies currently available to address this problem [5].

Microfiltration (MF) has been a promising separation technique for removing micron-sized particles such as bacteria, yeast cells and colloids through a porous membrane with pore sizes ranging from 0.1 μm to 10 μm due to its technical advantages such as gentle conditions, no phase change, the absence of additives and low energy expenditure, etc. [6,7].

Ceramic and polymeric MF membranes are the two main broad categories, both of which have their advantages and disadvantages [8]. Ceramic MF membranes exhibit excellent durability, and thermal and chemical stability, while they have less flexibility, lower productivity and a high cost [9,10]. Porous polymeric membranes manufactured by the very well-known phase inversion method are relatively inexpensive, but usually have their intrinsic limitations such as low flux and high-fouling performance due to the structural geometry of the pores, macro-void formation, the thickness factor and the corresponding pore size distribution [11,12,13,14]. Many studies have revealed that the nanofibrous membranes comprised of nanofibers with random orientation can overcome some of the limitations [15,16,17,18].

As a very promising way to achieve nanofibers, electrospinning has been paid considerable attention because it can produce micro- and nano-sized fibers through applying high electric fields [19,20,21]. The obtained nanofiber membranes usually have notable features such as large specific area, high porosity and an interconnected open pore structure [22,23,24,25]. These promising structural features make the nanofiber membrane a suitable candidate for filtration applications. Jang et al. [26] fabricated a PVDF/graphene oxide (GO) hybrid nanofiber membrane via electrospinning and the hybrid membrane exhibited high pure water flux. Ma et al. [27] reported a polyacrylonitrile (PAN) electrospun nanofibrous membrane which was surface-functionalized with poly (dual- and tri-vinyl monomers) and the membrane was demonstrated for effective removal of bacterial and viruses from contaminated water. Zhao et al. [8] constructed a composite microfiltration membrane, consisting of a PVDF asymmetric microporous membrane, ultrathin nanofibers and non-woven polyethylene terephthalate (PET) fabric, and the composite membrane showed a much better performance than the commercial microfiltration membrane. However, due to the relatively large pore size, the electrospun nanofiber membrane usually served as a mid-layer over a non-woven fabric support and was coated with a barrier layer in order to filter out minute objects by size exclusion [27,28], and the process is relatively complex.

Previous studies have shown that the reduced fiber diameter contributed to reducing the pore size [11]. Nanofibers with small pore size can be produced by modulating a combination of the solution and processing parameters (e.g., the polymer solution composition and concentration, applied voltage, collector distance and spinning rate) [29,30]. Wang et al. [31] prepared PA-66 spider-net nanofiber membranes via regulation of the solution properties and several electrospinning process parameters, and the spider-net nanofiber membrane exhibited small pore size and a better filtration performance. Wang et al. [32] reported scaffold-like structured polyacrylonitrile (PAN)/silica nanofiber membranes by regulating the jet number ratios of various concentrations of PAN solutions via multi-jet electrospinning, and the membrane showed excellent filtration properties. In our previous works, a novel polyvinylidene fluoride (PVDF) tree-like nanofiber membrane (PVDF-TLNM) was fabricated via one-step electrospinning for the first time [33]. Previous studies have demonstrated that the PVDF-TLNM exhibited small pore size, excellent mechanical properties and a good organic solvent–resistance performance. As we all know, PVDF is a material with extraordinary properties including easy moldability, good toughness, flexibility, and excellent chemical and thermal resistance, and it is commonly used in developing microfiltration and ultrafiltration membranes commercially [25,34].

In this contribution, the effect of tetrabutylammonium chlorid (TBAC) content on the morphology and pore size of nanofiber membranes was studied. Meanwhile, the hydrophilic properties of the microfiltration membrane were investigated. Furthermore, the microfiltration properties of the PVDF-TLNMs were evaluated. This study sheds new insight on the role of electrospinning nanofiber membranes as microfiltration membranes and may contribute greatly towards the development of better nanofiber membranes.

## 2. Experimental

### 2.1. Materials

PVDF (M_w_ = 520,000, Shanghai 3F New Materials Co., Ltd., Shanghai, China) was used as starting material. TBAC (Aladdin Co., Ltd., Shanghai, China, analytical grade) was used as additive. *N*,*N*-dimethylformamide (DMF) and acetone (Tianjin Kermel Co., Ltd., Tianjin, China, analytical grade) were used as solvents. All of the materials were used as received without further purification.

### 2.2. Fabrication of the Tree-Like Nanofibers

The PVDF/TBAC tree-like nanofiber mat was fabricated by adding certain amount of TBAC into PVDF solution via one-step electrospinning. The exhaustive methods were reported in our published paper [33]. Typically, PVDF solution at a concentration of 17 wt % was prepared by dissolving PVDF powder in DMF/acetone mixture at a ratio of 3/1 (v/v) by vigorous stirring. PVDF/TBAC solution was obtained by adding TBAC in the PVDF solutions with stirring for 1 h. The TBAC concentrations were 0.05, 0.10 and 0.15 mol·L^−1^, respectively. The electrospinning apparatus consisted of a micro syringe pump, a grounded collector and a high voltage supply set. The polymer solution was in a horizontally placed graduated pipette with a metal needle and then attached to a micro syringe pump. The electrospinning process was carried out with a voltage of 25 kV, tip-to-collector distance of 15 cm and spinning rate of 1 mL·h^−1^. As a control, we also prepared pure PVDF nanofiber membranes (PVDF-NMs). The nanofiber membranes were collected on the surface of a grounded aluminum foil and the nonwoven polypropylene (PP).

### 2.3. Characterization

A field emission scanning electron microscopy (FE-SEM) (S-4800, Hitachi Ltd., Tokyo, Japan) was used to observe the morphology of nanofiber membranes. The pore size and distributions of each nanofiber membranes were measured by pore size meter (PSM-165, Topas, GmbH, Frankfurt, Germany). The structural information of nanofiber was characterized by Fourier transform infrared spectroscopy (FT-IR) (TENSOR37, BRUKER, Dresden, Germany). The hydrophilicity performance of the membranes was evaluated on a static water contact angle measurement instrument (JYSP-180, Beijing Jinshengxin Testing Machine Co., Ltd., Beijing, China).

### 2.4. Microfiltration Evaluation

A dead-end filtration module equipped with a pressure control was used to measure the pure water flux and retention ratio of the nanofiber membranes. The pure water flux was defined as the volume of water passing through the membrane per unit time, per unit area and per unit of transmembrane pressure [35]. This property reflects the energy required to generate permeate and is an easy way to compare the permeability of different membranes. The tested membranes were cut as a disc shape with effective diameter of 25 mm and placed in the membrane cell. In this measurement, the membranes were pre-washed for 10 min at 15 psi, in order to remove all trapped air bubbles and open all the pores in the membranes [35]. All filtration data reported in this work without special descriptions were all obtained under the aforementioned conditions. Each experiment was conducted using one membrane disk. The distilled water was supplied across the membrane to test its water flux at different pressure. The micro-particle retention ratio of the nanofiber membrane was tested using the following procedure. Polystyrene (PS) microspheres of 0.3 μm in size were purchased from Tianjin University (Tianjin, China). Then 100 ppm suspensions of 0.3 μm PS microspheres were ultrasonic dispersed in distilled water for 30 min. Then a 250 mL suspension was filtered through the membranes at 7.2 psi.

The PS microspheres solution was detected by ultraviolet-visible (UV-Vis) spectroscopy. A calibration curve of concentration against absorbance of PS microspheres solution was plotted by varying concentration of filtrate solution, from which the concentration of filtrate solution was determined. The retention ratio was calculated by using the following equation [8].

(1) $$\text{Retention Ratio}\left(\%\right)=\frac{C_{f}-C_{p}}{C_{f}}\times 100\%$$

where $C_{f}$ and $C_{p}$ represent the PS microspheres concentration of the feed solution and that of the permeate solution, respectively.

## 3. Results and Discussion

### 3.1. Characterization of the Nanofiber Membranes

**Figure 1:**
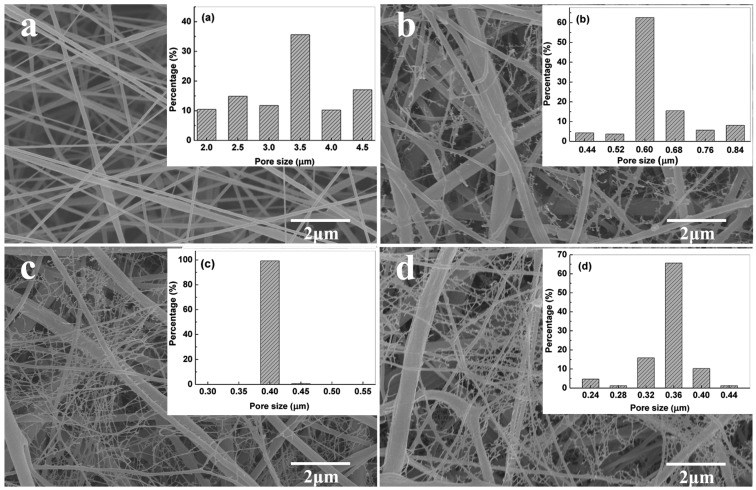
Field emission scanning electron microscopy (FE-SEM) images of polyvinylidene fluoride (PVDF) nanofiber membranes with different tetrabutylammonium chloride (TBAC) concentration: (**a**) no salt (pure PVDF); (**b**) 0.05 mol·L^−1^ polyvinylidene fluoride tree-like nanofiber membrane-1 (PVDF-TLNMs-1); (**c**) 0.10 mol·L^−1^ (PVDF-TLNMs-2); and (**d**) 0.15 mol·L^−1^ (PVDF-TLNMs-3) (the inset is the pore size distribution of the membrane).

**Figure 2:**
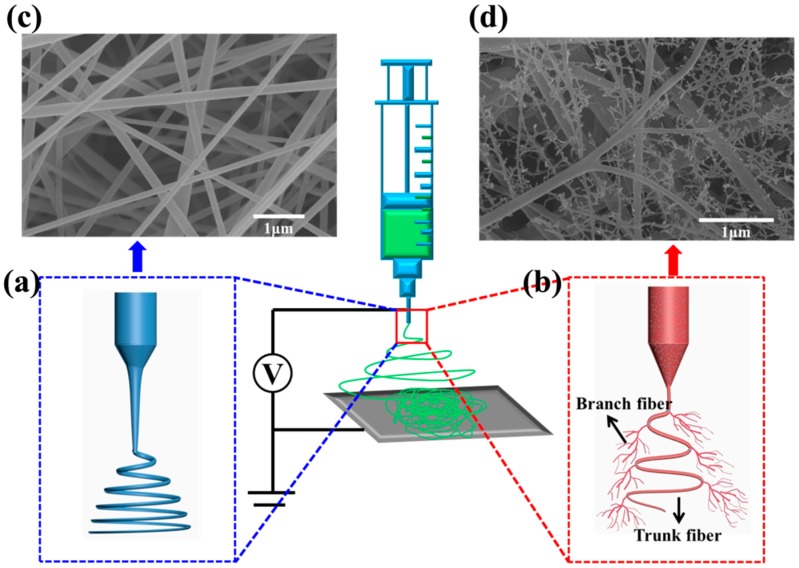
Schematic diagram for the electrospinning process of (**a**) pure PVDF solution and (**b**) PVDF/TBAC solution; (**c**) FE-SEM images of polyvinylidene fluoride nanofiber membranes (PVDF-NMs) and (**d**) PVDF-TLNMs.

Figure 1 presents the FE-SEM images of the PVDF-NMs and PVDF-TLNMs with different concentrations of TBAC. As can be seen from Figure 1a, the PVDF-NMs exhibit a conventional circular structure, with an average diameter of 180 nm. In contrast, the formation of large-scale tree-like nanofibers, which are composed of trunk fibers with a diameter of 100–500 nm and branch fibers with a diameter of 5–100 nm, can be observed from the membranes loaded with TBAC (Figure 1b–d). It was clear that the content of the tree-like structure increased with the increase of the TBAC concentration. This was because the increase of conductivity caused by the increase of the TBAC concentration was beneficial to the splitting of the charged jet [36,37]. The mechanism for the formation of the tree-like nanofibers has been reported in our previous paper [33]. Typically, for the electrospinning process of pure PVDF solution (Figure 2a), the jet followed a direct path on its way for certain a distance, and then whipped significantly to form a series of coils. However, for the electrospinning process of PVDF/TBAC (0.1 mol·L^−1^) solution (Figure 2b), there was a slight bending perturbation for the main jet from the spinning nozzle to the collector; meanwhile, some branching jets ejected from the main jet continually and further sub-divided into much thinner branches. At last, the main jet formed trunk fibers and the branching jet formed branch fibers. As can be seen from the inset images of Figure 1, the pore size of PVDF-TLNMs was much smaller than that of PVDF-NMs and the pore size of PVDF-TLNM nanofiber membranes decreased dramatically with the increase of the TBAC concentration. When the concentration of the TBAC increased from 0.05 to 0.15 mol·L^−1^, the pore size of the nanofiber membranes with a thickness of 30 μm decreased from 0.6 to 0.36 μm. The decreased pore size of the nanofiber membranes could be ascribed to the compact connections between the dense tree-like branch fibers. Meanwhile, the reduced fiber diameter contributed to the decrease of the pore size [11]. In addition, it was clear that the pore size distribution range of PVDF-TLNMs was narrower than that of PVDF-NMs, which made it a promising candidate in the field of microfiltration.

**Figure 3:**
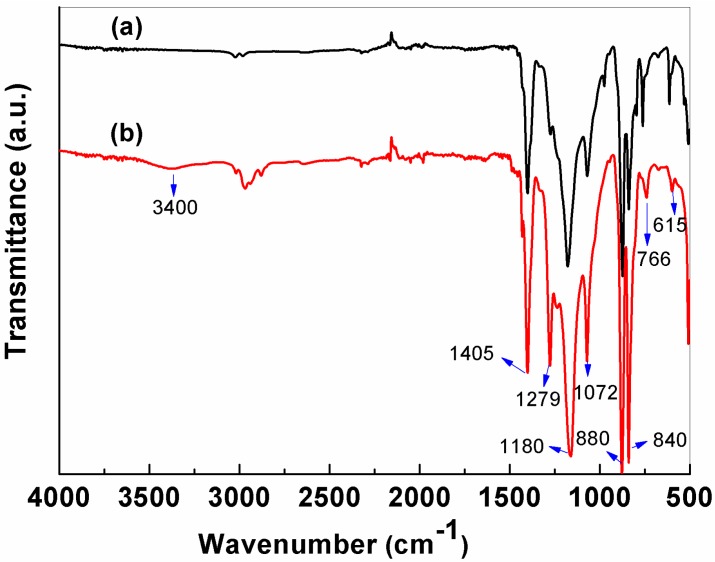
Fourier transform infrared spectroscopy (FT-IR) spectroscopy for: (**a**) PVDF-NMs; (**b**) PVDF-TLNMs-3.

Figure 3 shows the FT-IR spectroscopy results for the PVDF-NMs and PVDF-TLNMs-3. The bands occurring at 615 and 766 cm^−1^ were attributed to the CF_2_ bending vibration and the band at 1279 cm^−1^ was due to C-F stretching [38]. The bands appearing at 880, 1072, 1180, and 1405 cm^−1^ corresponded to CH_2_ in- and out-of-plane wagging or twisting, and the band appearing at 840 cm^−1^ was assigned to CH_2_ rocking [39]. It was obvious that the absorption band of CH_2_ was enhanced from the addition of the TBAC. In addition, a weak and broad absorption band at about 3400 cm^−1^ appeared after adding the TBAC, which was usually assigned to hydrogen-bonded N–H or O–H stretching vibration. However, as both PVDF and TBAC do not have N–H or O–H bonds, this broad peak is likely to be from the residue water in the membranes caused by the hygroscopic nature of the TBAC [40].

### 3.2. Hydrophilicity Measurements

**Figure 4:**
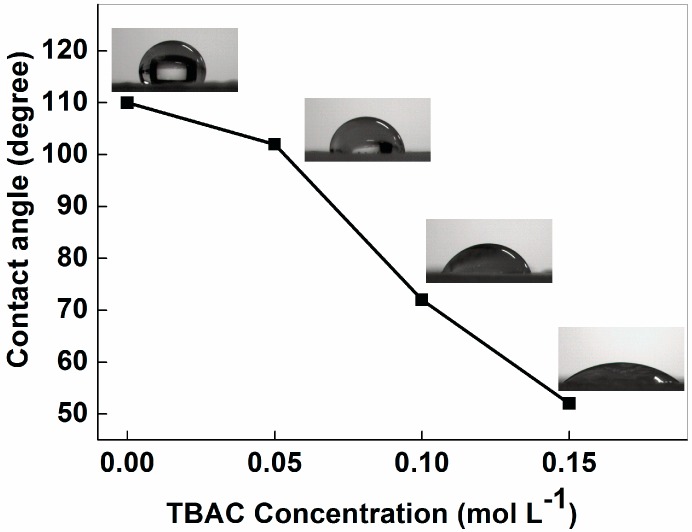
Contact angle of PVDF nanofiber membranes with different contents of TBAC.

For typical membrane filtration, the membranes with hydrophilic surfaces are less susceptible to fouling with organic substances, microorganisms, and charged inorganic particles due to a decrease in the interaction between the foulant and the membrane surface [37,41]. The hydrophobic nature of PVDF has severely limited its application in microfiltration. Figure 4 shows the water contact angle after a residence time of 1 s for PVDF nanofiber membranes with different concentrations of TBAC. As shown in this figure, in comparison with the PVDF-NMs, a sharp decrease in the contact angle could be observed from the PVDF-TLNMs. When the content of the TBAC increased from 0 to 0.15 mol·L^−1^, the contact angle decreased from 110° to 52°. Meanwhile, the contact angle of the PVDF-TLNMs was much smaller than that of the PVDF membranes prepared by phase inversion according to the literature, as shown in Table 1. Therefore, the addition of the TBAC had mutated the hydrophilic nature of the PVDF nanofiber membrane. This phenomenon is likely to be induced by the hygroscopic nature of the TBAC [40]. In addition, the increase of the hydrophilicity can be explained with the help of the increased surface energy. The increased surface area due to the presence of high-aspect-ratio tree-like nanofibers was responsible for the increase of the surface energy and consequentially increased the hydrophilicity [30].

### 3.3. Microfiltration Performance Evaluation

**Figure 5:**
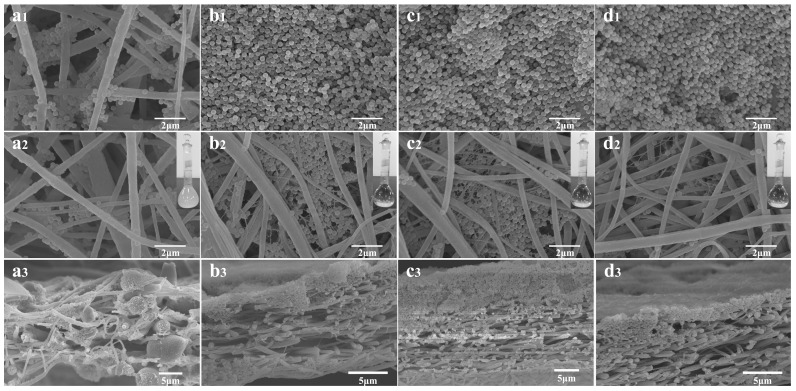
FE-SEM images of the top surface, bottom surface and cross-section of the membranes after a filtration test: (**a1**–**a3**) PVDF-NMs; (**b1**–**b3**) PVDF-TLNMs-1; (**c1**–**c3**) PVDF-TLNMs-2; (**d1**–**d3**) PVDF-TLNMs-3 (inset: photographs of the filtrate solutions).

As a proof of concept, we demonstrated that the membrane could be used for microfiltration applications. To evaluate the retention ratio of the PVDF-NMs and the PVDF-TLNMs with a thickness of 30 ± 2 μm, the thin electrospun membranes were deposited between the two layers of nonwoven PP substrates. We had measured the retention ratio of the nonwoven substrates. The result showed that the retention ratio of the nonwoven substrate was 0.75% which was negligible. Thus, the retention ratio measured across the composite mats can be approximated to the electrospun membranes. Then the monodispersed PS microspheres of 0.3 μm were passed through the composite membranes. A fresh membrane was replaced for each filtration. The results of the filtration are summarized in Table 2. The PVDF-NMs showed only a 46.29% retention ratio for the 0.3 μm PS microspheres suspension. However, for the PVDF-TLNMs, with the increase of the TBAC concentration (0.05, 0.10, 0.15 mol·L^−1^), the retention ratios were 92.3%, 98.8% and 99.9%, respectively, which indicated that the PVDF-TLNMs were successful in rejecting the particles at this size and the PVDF-TLNMs (0.15 mol·L^−1^) with the smallest pore size exhibited the best microfiltration performance. It is worth noting that the size of the PS microspheres is less than the mean pore size of the PVDF-TLNMs, so in theory the microspheres could enter or pass through the large pores. The results may be caused by the so-called “layer effect” [48]. It can be clearly observed that the PS particles were easily and efficiently sieved by the PVDF-TLNMs (Figure 5(b1,c1,d1)). This was because the PS microspheres were small and they were able to pack closely together, which reduced the effective pore size of the electrospun membrane significantly at the surface, leading to a “higher” retention ratio. In contrast, the PS particles passed through the PVDF-NMs directly and left little particles on the membrane surface (Figure 5(a1)). When the filtration performance of the PVDF-NMs and the PVDF-TLNMs was compared, it was clear that the particles were captured in the interior of the PVDF-NMs (Figure 5(a3)), whereas the particles were largely confined within the surface of the PVDF-TLNMs and only a very small fraction of the particles were found throughout the membrane thickness (Figure 5(b3,c3,d3)). It appeared that the electrospun PVDF-TLNMs (0.15 mol·L^−1^) were behaving as a screen filter in this instance.

**Figure 6:**
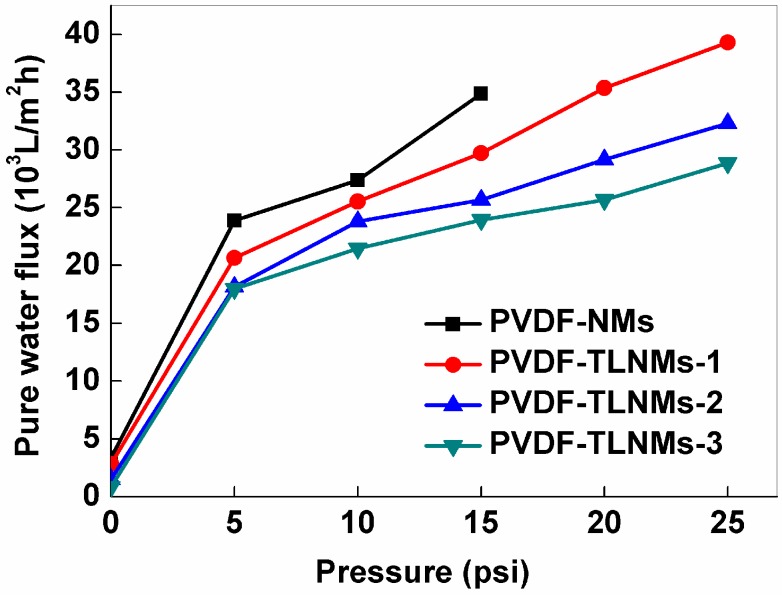
The pure water flux of PVDF-NMs, PVDF-TLNMs-1 (0.05 mol·L^−1^), PVDF-TLNMs-2 (0.10 mol·L^−1^) and PVDF-TLNMs-3 (0.15 mol·L^−1^).

The pure water flux was defined as the volume of water passing through the membrane per unit time and per unit area. The pure water flux of PVDF-NMs and PVDF-TLNMs with a thickness of 30 ± 2 μm was shown in Figure 6. The results indicated that the pure water flux was dependent on the pressure. With a larger pore size (see Table 2), the PVDF-NMs had a higher pure water flux than the PVDF-TLNMs. However, the pure water flux of the PVDF-TLNMs was significantly higher than that of the PVDF membranes prepared by the phase inversion method, as shown in Table 1. The results indicate that the PVDF-TLNMs could find potential application in a high-flux microfiltration membrane.

## 4. Conclusions

A novel PVDF tree-like nanofiber membrane with a small pore size and a narrow pore size distribution range was successfully fabricated by adding a certain amount of TBAC into the PVDF solution via electrospinning. The SEM images showed that the tree-like nanofibers were composed of trunk fibers with a diameter of 100–500 nm and branch fibers with a diameter of 5–100 nm. The hydrophilic properties of the PVDF-TLNMs were significantly improved. The PVDF-TLNMs (0.15 mol·L^−1^) showed a satisfactory retention ratio of over 99% against 0.3 μm PS particles and high pure water flux. The present study suggests that the PVDF-TLNMs have good potential for microfiltration applications. We will further demonstrate the long-term performance, durability, and regeneration tests of the PVDF-TLNMs in future studies.

## Figures and Tables

**Table 1:** Comparison of PVDF-TLNM and various membranes prepared by the phase inversion method.

Membranes (Optimum Parameter)	Contact Angle (°)	Water Flux (L/m^2^·h)	Pressure (MPa)	References
PVDF-TLNM	52	23,930	0.1	This study
PVDF/graphene oxide (GO)	60.5	324.5	0.025	[42]
PVDF-*g*-polyvinylpyrrolidone	62.65	200	0.01	[43]
PVDF-ZnO	70.06	452.1	0.05	[44]
PVDF/Mg(OH)_2_	76.2	2800	0.05	[45]
PVDF/dimethyl sulphoxide	81.2	272.2	0.1	[46]
SBA-15/PVDF	84	500	0.1	[47]

**Table 2:** The physical properties and filtration results for PVDF-NMs and PVDF-TLNMs.

Samples	TBAC Content (mol·L^−1^)	Largest Pore Size (μm)	Mean Pore Size (μm)	Smallest Pore Size (μm)	Retention Ratio (%)
PVDF-NMs	0	4.06	3.52	2.89	46.3
PVDF-TLNMs-1	0.05	0.82	0.60	0.41	92.3
PVDF-TLNMs-2	0.10	0.47	0.40	0.38	98.8
PVDF-TLNMs-3	0.15	0.41	0.36	0.22	99.9
